# Inequalities in Periodontal Disease According to Insurance Schemes in Thailand

**DOI:** 10.3390/ijerph18115945

**Published:** 2021-06-01

**Authors:** Jarassri Srinarupat, Akiko Oshiro, Takashi Zaitsu, Piyada Prasertsom, Kornkamol Niyomsilp, Yoko Kawaguchi, Jun Aida

**Affiliations:** 1 Department of Oral Health Promotion, Graduate School of Medical and Dental Sciences, Tokyo Medical and Dental University, Tokyo 113-8510, Japan; jarassri.ohp@tmd.ac.jp (J.S.); zaitsu.ohp@tmd.ac.jp (T.Z.); yoko.ohp@tmd.ac.jp (Y.K.); Aida.ohp@tmd.ac.jp (J.A.); 2 Bureau of Dental Health, Department of Health, Ministry of Public Health, Nonthaburi 11000, Thailand; piyada.p@anamai.mail.go.th (P.P.); kornkamol.n@anamai.mail.go.th (K.N.); 3 Division for Regional Community Development, Liaison Center for Innovative Dentistry, Graduate School of Dentistry, Tohoku University, Miyagi 980-8575, Japan

**Keywords:** inequalities, periodontal disease, insurance, National Oral Health Survey

## Abstract

Few studies have considered the effects of insurance on periodontal disease. We aimed to investigate the association between insurance schemes and periodontal disease among adults, using Thailand’s National Oral Health Survey (2017) data. A modified Community Periodontal Index was used to measure periodontal disease. Insurance schemes were categorized into the Universal Coverage Scheme (UCS), Civil Servant Medical Benefit Scheme (CSMBS), Social Security Scheme (SSS), and “others”. Poisson regression was applied to estimate the prevalence ratios (PRs) of insurance schemes for periodontal disease, with adjustment for age, gender, residential location, education attainment, and income. The data of 4534 participants (mean age, 39.6 ± 2.9 years; 2194 men, 2340 women) were analyzed. The proportions of participants with gingivitis or periodontitis were 87.6% and 25.9%, respectively. In covariate adjusted models, lowest education (PRs, 1.03; 95% CI, 1.01–1.06) and UCS (PRs, 1.05; 95% CI, 1.02–1.08) yielded significantly higher PRs for gingivitis, whereas lowest education (PRs, 1.20; 95% CI, 1.05–1.37) and UCS (PRs, 1.17; 95% CI, 1.02–1.34) yielded substantially higher PRs for periodontitis. Insurance schemes may be social predictors of periodontal disease. For better oral health, reduced insurance inequalities are required to increase access to regular dental visits and utilization in Thailand.

## 1. Introduction

Socioeconomic circumstances, including the health insurance system, affect oral diseases in both developing and developed countries [1]. Insurance is an important factor in improving access to healthcare. Access to healthcare is also influenced by socioeconomic circumstances, such as education and income [2]. However, few previous studies on social inequalities in oral health have considered the effects of insurance. This is because the health insurance schemes in many countries do not cover dental care as they do medical care [3]. Even in research on universal health coverage, dental care is rarely considered [4].

Although dental care is neglected in the context of universal healthcare, in some countries, a wider range of dental care is covered by public insurance schemes [5]. In Thailand, three public insurance schemes cover dental care. First, the Universal Coverage Scheme (UCS), which is free of charge, covers medical care, prescription drugs, and dental care. Approximately 70% of the population is covered by the UCS. Second, the Civil Servant Medical Benefit Scheme (CSMBS) accounts for approximately 9% of the Thai population, and covers government employees, retirees, and their family members. Both the UCS and CSMBS are tax-funded schemes. Both schemes provide comprehensive dental care comprising scaling or prophylaxis; restoration, not including esthetic or endodontic treatment in posterior teeth; and extraction and surgical removal. For prosthetic treatment, people can insert a removable denture which has been covered by insurance scheme for 5 years. Third, the Social Security Scheme (SSS) is a mixed-funded scheme (copayment or coinsurance). Scaling or prophylaxis, restoration, extraction and surgical removal are all covered by SSS for 900 Baht per year (approximately 28.74 USD). Otherwise, it is the responsibility of the payer to continue to separately pay for dental facilities and other providers. Sixteen percent of the population who are private-sector employees use the SSS [6,7]. 

Periodontal disease accounts for one-third of the global burden of oral disease. Particularly in developing countries, studies have reported a high prevalence of gingivitis among most children and adults [8]. Therefore, periodontitis is the main cause of tooth loss and affects the masticatory function, esthetics, and quality of life [9]. The evidence from a 26-year follow-up study has clearly shown that gingivitis is a precursor of periodontitis, and areas affected by long-standing gingivitis have a higher incidence of periodontitis [8]. Previous studies showed that socioeconomic status is associated with the occurrence of periodontal diseases [10,11,12]. Low education level, and lack of regular dental visits in older adults worsen the progression of periodontal disease [10]. To reduce social disparities and improve access to periodontal treatment, universal dental coverage and changes in dental policies at the national level should be considered [13,14]. However, few studies mention inequalities and insurance schemes in relation to periodontal disease. The objective of the present study was to investigate the association between social circumstances and the occurrence of periodontal disease, by considering insurance schemes among adults, using data from Thailand’s National Oral Health Survey. 

## 2. Materials and Methods

### 2.1. Ethical Considerations

The study protocol was approved by the ethics committees of the Department of Health, Ministry of Public Health, Thailand (approval date, 19 September 2019, no. 353; extended approval date, 10 September 2020, no. RF 13-01-353) and the Tokyo Medical and Dental University (approval no. D2019-057). The study was conducted in accordance with the Declaration of Helsinki put forth by the World Medical Association.

### 2.2. Setting and Participants

In the present cross-sectional study, we performed a secondary analysis of data from the eighth Thailand’s National Oral Health Survey. The national survey has been conducted every five years since 1977 by the Bureau of Dental Health, Department of Health, Ministry of Public Health, Thailand. The latest (eighth) survey was conducted from June to September, 2017. A stratified, three-stage sampling technique was used to represent the country. Following WHO’s oral health surveys methods, the target of Thailand’s National Oral Health Survey was separated by age groups; preschool children (3 and 5 years old), children and youth (12 and 15 years old), middle aged adults (35–44 years old), older adults (60–74 years old), and late older adults (80–85 years old). The target population from these indexed age groups were selected from 24 provinces in 12 health regions and the Bangkok Metropolitan Region, using systematic and quota sampling. Finally, 26,259 persons (49.4% males, 50.6% females) in all indexed age groups participated in this survey. After obtaining written informed consent from the participants, a structural questionnaire survey was conducted before clinical oral examination. Detailed information about the 8th Thailand’s National Oral Health Survey has been described elsewhere [15,16]. In the 8th survey, 4683 adults (aged 35–44 years) underwent oral examination and interviews. As periodontal disease is a prevalent health condition among middle-aged adults, the present analysis targeted this age group. The periodontal status was assessed via oral examination, using the WHO periodontal probe and a plane mouth mirror. To ensure the reliability and validity of the data, intra-examiner and inter-examiner reproducibility was assessed among 19 dentists who were trained and practiced under standardized conditions during the calibration period, in accordance with the WHO protocol [17]. The Kappa score for periodontal status was 0.41–0.66, demonstrating a moderate agreement level. 

### 2.3. Dependent Variables

The modified Community Periodontal Index (CPI) was used to obtain gingival bleeding and pocket scores. Thailand’s National Oral Health Survey applied a more detailed classification of periodontal status than the WHO coding system [17]. The presence or absence of gingivitis or periodontitis was evaluated and used as the dependent variable in this study. As a dependent variable for gingivitis, the gingival bleeding score was classified into an absence of the condition (WHO code 0) or a presence of the condition (WHO codes 1, 2, and 5). As a dependent variable of periodontitis, the pocket score was classified into an absence of the condition (WHO code 0) or a presence of the condition (WHO codes 1 and 2, mean pocket depth ≥4 mm). Both gingival bleeding and pocket scores and the data of participants with code 9 or X (tooth excluded or not present) were excluded from our analysis.

### 2.4. Independent Variables

Participants were asked to respond to questions regarding each of the variables discussed below.

#### 2.4.1. Demographic Information

We assessed data regarding age group (35–39 years; 40–44 years) and sex (‘men’, ‘women’). We also used residential areas as demographic covariates. Based on the areas where the participants lived, the residential area was categorized into ‘rural’ or ‘urban’ areas.

#### 2.4.2. Socio-Economic Status

We considered educational attainment and income as measures of socioeconomic status. For educational attainment, the participants were asked to select the highest level of education obtained. The responses were categorized as follows—lowest (≤6 years), moderate (7–9 years), high (10–12 years), and highest (≥13 years) education. The income per month was divided into four categories, and we combined the categories as follows lowest (≤159.69 USD), moderate (159.72–479.08 USD), high (479.11–958.16 USD), and the highest (≥958.19 USD) income at an exchange rate of 1 USD = 31.31 THB.

#### 2.4.3. Health Insurance Schemes

We enquired about insurance schemes with 10 choices, and we combined the responses under the UCS, CSMBS, SSS, and others (do not use, do not have, and do not know). All residents in Thailand had insurance. Therefore, those who responded “do not have” were combined into “others.”

### 2.5. Statistical Analysis

We cross-tabulated the dependent and independent variables using the Chi-square test. We applied Poisson regression with robust variance because the prevalence of periodontal disease in the present study was not low. Prevalence ratios (PRs) and 95% confidence intervals (CI) were applied instead of odds ratios for gingivitis or periodontitis, which would be overestimated [18,19,20,21]. First, the univariate PRs was calculated for each independent variable. Demographic covariates (age, gender, and residential area) were then adjusted. To avoid the problem of over adjustment, data regarding education, income, and insurance schemes were separately included in the models, with adjustment for the covariates, because there were correlations among education, income, and insurance schemes. All statistical analyses were performed using SPSS version 22.0 (IBM Corporation, Tokyo, Japan). Analysis items with *p* < 0.05 were considered to be statistically significant.

## 3. Results

After excluding all missing data, a total of 4534 participants (2194 men; 2340 women) were included in this study. The mean age of the participants was 39.6 ± 2.9 years. The proportion of participants with gingivitis or periodontitis was 87.6% and 25.9%, respectively. Participants who lived in rural areas, had lower education levels, lower income, and were on the UCS showed significantly higher PRs for both gingivitis and periodontitis (Table 1).

In the covariate-adjusted models, participants with the lowest education (PRs, 1.03; 95% CI, 1.01–1.06) and UCS (PRs, 1.05; 95% CI, 1.02–1.08) had significantly higher PRs for gingivitis (Table 2). Table 3 shows the trend in PRs among participants was similar to that of gingivitis. Participants with the lowest education level (PRs, 1.20; 95% CI, 1.05–1.37) and UCS (PRs, 1.17; 95% CI: 1.02–1.34) had substantially higher PRs for periodontitis in the covariate-adjusted models.

## 4. Discussion

The present study determined the association between insurance schemes and the occurrence of periodontal disease in the adult population in Thailand. To the best of our knowledge, the results indicated a significantly higher prevalence of gingivitis and periodontitis among insurance schemes, even after adjusting for relevant confounding variables. The degrees of these associations were compared to that of education. The present study suggests that insurance schemes are important predictors of periodontal disease.

Previous studies conducted in many countries have also reported that insurance systems are important predictors of periodontal health and use of dental care among adults [6]. People with dental benefits or insurance had more dental visits or check-ups than those without any insurance in the United States [22]. In addition, insurance in Japan provides the discount of co-payment for dental treatments, which increases the frequency of both treatments and check-up visits. Insurance ensuring access to necessary dental treatments and basic preventive dental care nationwide is required to decrease the incidence of periodontal disease [14,23]. In South Korea, the public health insurance provides annual dental scaling for the prevention and management of periodontal diseases. Although this policy did not eliminate oral health inequalities, it improved access to preventive dental care [13].

A systematic review also reported that subjects with lower educational attainment had a higher risk of periodontitis [24]. An association between income and the occurrence of periodontitis has also been reported [25,26]. In the present study, social inequalities and education level showed a higher association with periodontal disease than income. Inequalities in oral healthcare are present right from the early stages of life [27]. Access to education can indicate socioeconomic status at a younger age [28]. Previous studies have showed that a higher prevalence of periodontal disease was related to lower educational attainment, as compared to higher education [29]. Furthermore, a previous study showed that participants with high education level and middle-class income who received regular dental care during early life could maintain oral health status with good function throughout their lives [30]. Therefore, it is reasonable that an effect of education as a proxy of social inequality on periodontal disease was observed in the present study. In contrast, the effect of income as a proxy of social inequalities on periodontal disease was weak. This could be attributable to the fact that the level of insurance support, assessed via income inequalities in relation to access to dental care in Thailand was lower than those of inequalities regarding educational attainment. 

Our practical evidence implies the importance of insurance schemes on the occurrence of periodontal disease; adults with UCS had a higher risk of periodontal disease. In Thailand, the National Health Security Office established dental benefits packages in the UCS, including regular dental check-ups and preventive programs for high-risk groups. However, participants with UCS are less likely to utilize dental care [31]. A previous study conducted in Thailand reported that elderly participants insured under the CSMBS had 1.3 times significantly higher chance of visiting dental services than people insured under the UCS [16]. One possible reason for the lower level of access could be due to the long waiting time for dental care, because the provision of dental care under the UCS is delivered only during the office hours in public dental facilities. A randomized control trial conducted in Germany reported that a difference in waiting time due to insurance schemes affected healthcare access [32]. In addition, a long waiting time can worsen the quality of dental care. Certain dental treatment procedures require several appointments. Under the UCS, long waiting times impose longer intervals between visits among patients. These rather long intervals among multiple treatment procedures could reduce access to dental care. To solve this problem via coordination between the public and private sectors, it is necessary to expand the UCS coverage from public facilities to private dental clinics. In Japan, a wider range of dental treatments in both private and public dental clinics is covered by public insurance [33,34].

The cost of dental care is also considered to impede access to dental care. A previous study reported that changes in insurance coverage due to the COVID-19 pandemic affected the pattern of access to dental care [35]. Increments in costs have reduced access to routine dental check-ups and increased emergency use for dental problems. In our study, the SSS and “other” insurance schemes did not show any significant difference in the occurrence of periodontal disease, as compared to the CSMBS. Participants insured under the SSS and “other” insurance schemes had to pay for dental treatments, whereas participants insured under the UCS and CSMBS did not need to pay for treatments [7]. Therefore, our results support the idea that participants insured under the SSS and “other” insurance schemes also had a higher risk of periodontal disease. This discrepancy may be explained by the level of educational attainment. People insured under the SSS and “other” insurance schemes could have a higher educational attainment than those insured under the UCS. 

The most important strength of our study was that it was a large epidemiological survey that represented the adult population of Thailand in terms of socioeconomic circumstances, including the insurance system and periodontal status. As a limitation, however, our study was a cross-sectional study that failed to establish a causal relationship between the independent variables and outcomes. Therefore, further longitudinal studies are required to assess these.

## 5. Conclusions

After adjusting for age, gender, and residential location, participants insured under the UCS had a significantly higher risk of periodontal disease. Insurance schemes may be social predictors of periodontal disease. There is a need to reduce barriers to dental care access among adults with different healthcare insurance schemes in Thailand.

## Figures and Tables

**Table 1:** Distribution of gingivitis and periodontitis among adults aged 35–44 years in Thailand.

	*n* = 4534 *n* (%)	Gingivitis		*p*-Value *	Periodontitis		*p*-Value *
Variable		Absence of Condition, Code 0 (*n* = 560)	Presence of Condition, Code 1, 2, or 5 (*n* = 3974)		Absence of Condition, Code 0 (*n* = 3359)	Presence of Condition, Code 1 or 2 (*n* = 1175)	
**Sociodemographic characteristics**							
**Age, years**				0.571			0.776
35–39	2245 (49.5)	271 (12.1)	1974 (87.9)		1659 (73.9)	586 (26.1)	
40–44	2289 (50.5)	289 (12.6)	2000 (87.4)		1700 (74.3)	589 (25.7)	
**Gender**				0.653			0.915
Male	2194 (48.4)	266 (12.1)	1928 (87.9)		1627 (74.2)	567 (25.8)	
Female	2340 (51.6)	294 (12.6)	2046 (87.4)		1732 (74.0)	608 (26.0)	
**Location**				**<0.001**			**<0.001**
Urban	2260 (49.8)	328 (14.5)	1932 (85.5)		1768 (78.2)	492 (21.8)	
Rural	2274 (50.2)	232 (10.2)	2042 (89.8)		1591 (70.0)	683 (30.0)	
**Education attainment**				**0.028**			**0.001**
Lowest (≤6 years)	1180 (26.0)	119 (10.1)	1061 (89.9)		823 (69.7)	357 (30.3)	
Moderate (7–9 years)	647 (14.3)	76 (11.7)	571 (88.3)		476 (73.6)	171 (26.4)	
High (10–12 years)	1344 (29.6)	179 (13.3)	1165 (86.7)		1020 (75.9)	324 (24.1)	
Highest (≥13 years)	1363 (30.1)	186 (13.3)	1177 (86.4)		1040 (76.3)	323 (23.7)	
**Income (per month)**				**0.012**			**0.037**
Lowest (≤159.69 USD)	872 (19.2)	80 (9.2)	792 (90.8)		634 (72.7)	238 (27.3)	
Moderate(159.72–479.08 USD)	2238 (49.4)	284 (12.7)	1954 (87.3)		1631 (72.9)	607 (27.1)	
High (479.11–958.16 USD)	1067 (23.5)	147 (13.8)	920 (86.2)		824 (77.2)	243 (22.8)	
Highest (≥958.19 USD)	357 (7.9)	49 (13.7)	308 (86.3)		270 (75.6)	87 (24.4)	
**Insurance**				**<0.001**			**0.003**
Universal Coverage Scheme	2150 (47.4)	220 (10.2)	1930 (89.8)		1539 (71.6)	611 (28.4)	
Civil Servant Medical Benefit Scheme	934 (20.6)	140 (15.0)	794 (85.0)		721 (77.2)	213 (22.8)	
Social Security Scheme	1359 (30.0)	189 (13.9)	1170 (86.1)		1032 (75.9)	327 (24.1)	
Others	91 (2.0)	11 (12.1)	80 (87.9)		67 (73.6)	24 (26.4)	

* *p*-Values were calculated using the Chi-squared test.

**Table 2:** Association of educational attainment, income, and health insurance with the presence of gingivitis assessed via Poisson regression among adults aged 35–44 years in Thailand.

Variable	Total (*n* = 4534)	Univariate PRs	95% CI	Covariate Adjusted PRs *	95% CI
**Educational attainment**					
Highest (≥13 years)	1363	**Reference**		**Reference**	
High (10–12 years)	1344	1.00	0.97–1.03	1.00	0.97–1.03
Moderate (7–9 years)	647	1.02	0.99–1.06	1.01	1.00–1.06
Lowest (≤6 years)	1180	1.04	1.01–1.07	1.03	1.01–1.06
**Income (per month)**					
Highest (≥958.19 USD)	357	**Reference**		**Reference**	
High (479.11–958.16 USD)	1067	1.00	0.95–1.05	0.99	0.95–1.05
Moderate (159.72–479.08 USD)	2238	1.01	0.97–1.06	1.01	0.96–1.05
Lowest (≤159.69 USD)	872	1.05	1.01–1.10	1.04	1.00–1.09
**Insurance**					
Civil Servant Medical Benefit Scheme	934	**Reference**		**Reference**	
Social Security Scheme	1359	1.01	0.98–1.05	1.01	0.97–1.04
Universal Coverage Scheme	2150	1.06	1.02–1.09	1.05	1.02–1.08
Others	91	1.03	0.95–1.12	1.03	0.95–1.12

* Each socioeconomic status variable (educational attainment, income, and insurance) was separately included in the models, with adjustment for age, gender, and residential location. PRs—prevalence ratios; CI—confidence interval.

**Table 3:** Association of education, income, and insurance with the presence of periodontitis (pocket depth ≥ 4 mm) assessed via Poisson regression among adults aged 35–44 years in Thailand.

Variable	Total (*n* = 4534)	Univariate PRs	95% CI	Covariate Adjusted PRs *	95% CI
**Educational attainment**					
Highest (≥13 years)	1363	**Reference**		**Reference**	
High (10–12 years)	1344	1.02	0.89–1.16	0.99	0.86–1.13
Moderate (7–9 years)	647	1.11	0.95–1.31	1.05	0.90–1.24
Lowest (≤6 years)	1180	1.28	1.12–1.45	1.20	1.05–1.37
**Income (per month)**					
Highest (≥958.19 USD)	357	**Reference**		**Reference**	
High (479.11–958.16 USD)	1067	0.93	0.75–1.16	0.95	0.77–1.17
Moderate (159.72–479.08 USD)	2238	1.11	0.92–1.35	1.08	0.89–1.32
Lowest (≤159.69 USD)	872	1.12	0.91–1.38	1.06	0.86–1.32
**Insurance**					
Civil Servant Medical Benefit Scheme	934	**Reference**		**Reference**	
Social Security Scheme	1359	1.05	0.91–1.23	1.03	0.89–1.20
Universal Coverage Scheme	2150	1.25	1.09–1.43	1.17	1.02–1.34
Others	91	1.16	0.80–1.66	1.16	0.81–1.66

* Each socioeconomic status variable (educational attainment, income, and insurance) was separately included in the models, with adjustment for age, gender, and residential location. PRs—prevalence ratios; CI—confidence interval.

## Data Availability

The datasets presented in this study and on which analyses were conducted are available from the corresponding author on reasonable request.
